# Fabrication of polymer blend vascular grafts with enhanced mechanical properties and rapid cell infiltration: influence of micro/nanostructure, polymer composition, and post-processing on pore architecture and bioengineered environment

**DOI:** 10.1088/1748-605X/ae164d

**Published:** 2025-10-31

**Authors:** Aurora Battistella, Morgan Linger, Richard D Johnson, Meredith Overton, Anna Sallee, Rajan Jain, Bridget Antreasian, Yifu Ding, Wei Tan

**Affiliations:** 1 Paul M Rady Mechanical Engineering, University of Colorado at Boulder, Boulder, CO, United States of America; 2 Biomedical Engineering Program, University of Colorado at Boulder, Boulder, CO, United States of America

**Keywords:** microfiber, vascular graft, cell infiltration

## Abstract

Arteriovenous (AV) shunts are critical conduits for patients with end-stage renal disease undergoing hemodialysis. Desired properties of next-generation AV graft materials include artery-like mechanics, clinically feasible manufacturing processes, and a bioactive interface that facilitates rapid and deep infiltration of neighboring cells to support tissue regeneration. These requirements inspired the design, fabrication, and post-processing of our constructs. In terms of material design, we evaluated the performance of three microfiber graft materials composed of a hydrophobic polymer and photo-clickable, 4-arm thiolated polyethylene glycol-norbornene (PEG-NB). The materials included two coaxially nanostructured fiber designs, each featuring a PEG-NB sheath and different cores—polycaprolactone (PCL) and PCL-co-lactic acid (PLCL), respectively—and a mixed composition created by directly blending the sheath and core solutions during electrospinning. For post-processing, the constructs were either air-dried or freeze-dried (FD). Surface morphology was assessed using scanning electron microscopy, while mechanical properties were characterized through tensile testing and dynamic mechanical analysis. Subcutaneous implants were evaluated at 1, 4, and 16 weeks using histological, immunofluorescent, and multiphoton microscopy analyses to examine cellular distribution, material structure, and tissue remodeling. Results showed that the freeze-drying post-processing method enhanced overall porosity, stiffness, and ultimate tensile strength. Among all tested conditions, the FD core-sheath structure with PCL most closely matched the mechanical properties of native vessels. Using PLCL as a core material increased degradation and cell infiltration during the first month of subcutaneous studies. Ultimately, graft strength, porosity, and bioactivity were effectively modulated by the choice of core material and post-processing method. These findings provide insights into tailoring electrospun PEG-NB hybrid constructs as candidate AV shunt grafts, highlighting opportunities to balance mechanical performance, degradation, and bioactivity for end-stage renal disease patients requiring durable hemodialysis access.

## 1. Introduction

Cardiovascular diseases remain the leading cause of mortality worldwide, underscoring the critical need for innovative treatment and management strategies [1]. Among these, the demand for small-caliber vascular grafts (<6 mm) has driven extensive research into the development of new graft preparation methods [2–4]. In particular, arteriovenous (AV) grafts are urgently needed for hemodialysis access for patients with end-stage renal disease (ESRD) [5], as current options such as expanded polytetrafluoroethylene (ePTFE) are prone to thrombosis, intimal hyperplasia, and poor long-term patency [6].

While the specific clinical demands differ, such as the repeated cannulation and high flow conditions unique to AV grafts, the underlying challenge across all settings is to create a graft that combines mechanical durability with host integration and hemocompatibility [7, 8]. Current graft materials often fail to achieve satisfactory long-term efficacy, with complications such as stenosis, thrombosis, and infection contributing to high failure rates [9]. These issues are largely attributed to the stiff, bioinert properties of artificial grafts [10].

To address these limitations, tissue engineering has emerged as a promising solution, focusing on the design of biomimetic scaffolds that promote tissue regeneration and provide durable, functional conduits [11]. An ideal graft for *in situ* tissue engineering should be readily available for implantation, compliant, durable, and biologically active to support arterial regeneration and ensure long-term patency [12]. However, significant challenges remain in creating grafts that satisfy all these critical requirements while addressing biomanufacturing considerations, including aseptic processing and logistical constraints related to time and cost [13].

A critical aspect of graft development for *in situ* tissue engineering is the selection of scaffolding polymers, which provide structural support and mechanical stability [14]. The mechanical properties of these polymers are essential for maintaining scaffold integrity under physiological conditions, while their degradation behavior significantly impacts the scaffold’s capacity to support tissue regeneration over time [14]. Polycaprolactone (PCL) has been widely studied for its strength and slow degradation properties [15],whereas PCL-co-lactic acid (PLCL) is gaining attention as an alternative due to its faster degradation rate and higher elasticity, aimed at accelerating the *in situ* tissue remodeling process [16]. However, both polymers lack inherent bioactivity.

To overcome this limitation, our previous studies have utilized a versatile hydrogel material, 4-arm, end-functionalized polyethylene glycol-norbornene (PEG-NB) [17]. This material’s ability to bind various cell-adhesive and functional ligands via photoclickable ‘thiol-ene’ chemistry makes it an excellent candidate for bioengineered scaffolds that regulate cellular activities. We employed coaxial electrospinning to combine PCL with PEG-NB, creating nanostructured fibers that form a porous hybrid graft scaffold [17]. While these scaffolds are mechanically strong enough to function as native arteries, further advancements in graft degradation, compliance, and cell penetration are necessary to progress such hybrid grafts into vascular surgery.

In this study, we extend our prior work by directly comparing PCL-PEGNB and PLCL-PEGNB coaxial scaffolds, as well as blended formulations, with an emphasis on their suitability for AV shunt applications. Given the critical role of microstructural features such as porosity in cell penetration and tissue integration [18–20] and the substantial influence of post-processing on these properties, we further examined how air-drying and lyophilization alter scaffold structure, mechanics, and host response. Through this systematic evaluation of polymer selection, scaffold design, and processing impact graft performance, our goal is to inform the development of next-generation AV shunt grafts that integrate mechanical durability with bioactivity and remodeling potential to better serve ESRD patients.

## 2. Materials and methods

### 2.1. Materials

Unless specified otherwise, all polymers and chemicals were purchased from Sigma–Aldrich Inc. (St Louis, MO). PEG-NB (molecular weight 5000 Da) from Biopharma PEG Scientific Inc (Watertown, Massachusetts, PLCL (molecular weight 75 000–85 000 Da) from Polyscitech, a division of Akina Inc (West Lafayette, IN), and photoinitiator Irgacure-2959 was purchased from Ciba Specialty Chemicals (Basel, Switzerland).

### 2.2. Graft fabrication

The employed electrospinning apparatus was developed in-house. Coaxial fibers were obtained by a core hydrophobic polymer solution pumped through the inner 22 G needle and a sheath hydrophilic polymer solution pumped through the outer 18 G needle. The core solution is 5 wt% PCL (molecular weight 70 000–90 000 Da) or PLCL in Hexafluoroisopropanol, whereas the sheath solution contains 5.268 wt% 4-arm PEG-NB, 1.75 wt% PEG dithiol (PEG-SH, molecular weight 1000 Da), and 0.8 wt% polyethylene oxide (molecular weight 400 000 Da). The solution is allowed to dissolve overnight. Then, Irgacure-2959 is added as a photoinitiator; from this point on, the sheath solution needs to be protected from light. After preparing the two solutions, the dual syringe holder allows both syringes to be loaded and extruded simultaneously. The solutions were loaded in 5 ml syringes connected to the positive terminal of a high voltage ES30P 10 W power supply (Gamma High Voltage Research, Ormond Beach, FL). The core polymer solution was extruded at 0.8 mL h^−1^ and the sheath polymer solution at 1 ml h^−1^ using syringe pumps (Pump 11 Plus, Harvard Apparatus, Boston, MA) and subjected to an electric potential of 13 kV for approximately 2.5 h. The miscibility of the two solutions (hydrophobic and hydrophilic polymers) was checked and the 1:1 mixture was employed for mixed conditions, which were extruded at 1 mL h^−1^ through the 22 G needle.

Biological moieties were not added for materials characterization tests since they do not play a significant role in structural properties. They were added for *in vivo* experiments and platelet adhesion studies: diallyl trisulfide was added to the core solution, and CGRGDS peptide (GenScript, Piscataway, NJ) and Biotin-QK-VEGF express peptide (GenScript) were added to the sheath solution.

The fibers were deposited onto a grounded static aluminum substrate placed 15 cm perpendicular to the needle, and rectangular-shaped samples were obtained for mechanical tests. Circular glass slides were treated with (3-Mercaptoprropyl) trimethoxy silane (MPTS, TCI, Japan) for better adhesion of the collected fibers. The slides were placed on top of the aluminum base layer and adopted as a substrate for platelet adhesion tests and morphological observation via scanning electron microscopy (SEM). A square Electron Microscopy Sciences (Hatfield, PA) grid 200 mesh was used to collect fibers for transmission electron microscopy (TEM) measurement. For subcutaneous implantation, PTFE (RT Dygert, Burnsville, MN) rings were employed as base support since the frame allows a better retrieval of the sample during explant.

### 2.3. Post-processing techniques

**Figure 1.**
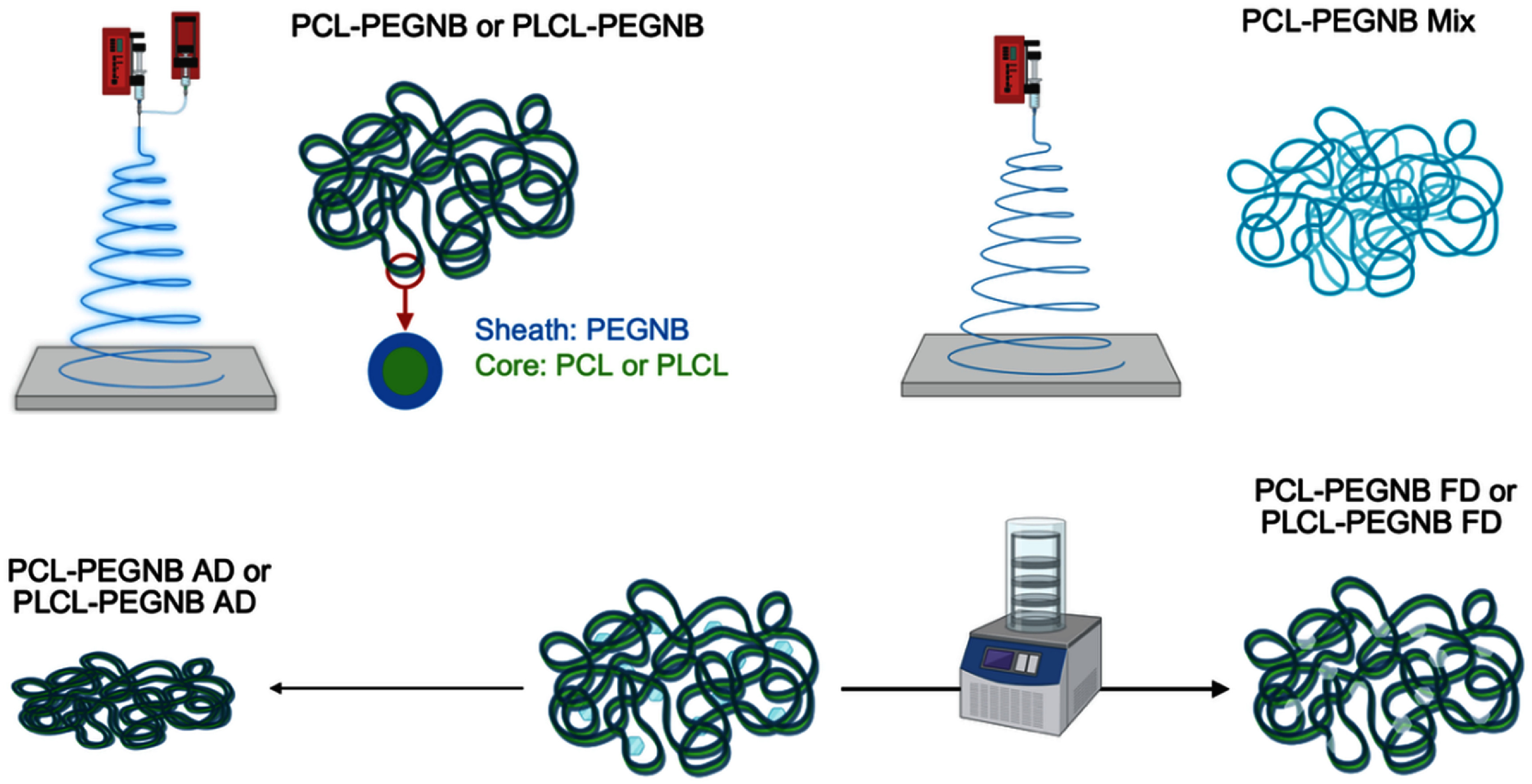
Illustration of sample preparation, showing fabrication (Top) and post-processing (Bottom).

The prepared samples were placed under vacuum for 15 min in a glovebox (Vacuum Atmospheres Company, OMNI-LAB, Hawthorne, CA) filled with argon to remove oxygen. Following this, the fiber mats were subjected to UV light for 90 min to initiate crosslinking. The crosslinking process involved a thiol-ene photo-click reaction, where the ‘ene’ groups in the 4-arm PEG-NB reacted with the thiol groups in the PEG-SH cross-linker. After cross-linking, the samples were immersed in phosphate-buffered saline (PBS, pH 7.4) for 24 h. Post-immersion, the samples were either air-dried (AD) by leaving them under a hood for 24 h or freeze-dried (FD) by rapidly freezing them in liquid nitrogen and then placed in a 700 401 000 FreeZone 4.5 l−50 °C Benchtop Freeze Dryer (Labconco, Kansas City, MO) for 24 h. An overview of the fabrication workflow, post-production treatments, and experimental groups under study is illustrated in figure 1.

### 2.4. Electron microscopy imaging of fiber morphology and scaffold structure

Electrospun fibers were imaged with SEM and TEM. To investigate fiber morphology in the scaffold, the MPTS-treated glass slides coated with the electrospun fibers were mounted on brass stubs and Pd/Pt sputter-coated using a Cressington sputter coater 108auto with a rotary vane vacuum pump VRL 100–3.5 (Cressington Scientific Instruments, Watford, UK). Observations were made using SEM (Hitachi SU3500 VP, Japan) at the Colorado Shared Instrumentation in Nanofabrication and Characterization (COSINC). Images were taken in three different locations of each sample to analyze the average pore size and fiber dimension, and measurements were averaged using Fiji ImageJ software. Fiber diameter was calculated only on PCL-PEGNB groups due to the loss of the fibrous network of PLCL-PEGNB samples after soaking in PBS. Calculating the pore size of the fibrous network of PCL-PEGNB groups was more challenging due to the different planes in which fibers lay. Therefore, the images were binarized, and the threshold was adjusted before measuring the pore dimension. TEM was used to image the nanostructure of all fibers. A FEI TecnaiTM T12 SpiritBT (FEI, Hillsboro, OR) was used with a CCD camera AMT XR41, operated at 100 kV.

### 2.5. X-ray photoelectron spectroscopy (XPS)

XPS was adopted to analyze the surface of materials to better understand the chemical and physical interactions between polymers. This technique allows us to investigate the coaxial structure with the assumption that the penetration depth of XPS is lower than the sheath thickness. Therefore, the XPS results should only report PEG-NB in the coaxial conditions. Instead, in the mixed-condition fibers, which were made by a blend of PCL or PLCL and PEG-NB, both polymers should be visible in the scans. No differences between PCL-PEGNB and PLCL-PEGNB specimens were expected, but differences were expected between coaxial and mixed samples. To mask the silica and oxygen signals from the glass slide substrate, a 20 nm gold coating was adopted. Survey and high-resolution 1Cs scans were performed.

### 2.6. Mechanical testing

Samples were electrospun onto a flat sheet of foil for 2 h for tensile tests and 6 h for dynamic mechanical analysis (DMA). For tensile testing, the samples were cut into 30 mm long x 6 mm wide rectangles and then shaped into dog bones by 2 mm cutouts on both sides. For DMA, specimens 5 mm in width and 12 mm in length were used. The ends of the specimens were sealed between plexiglass slides using superglue (Loctite). Samples were hydrated in PBS for 24 h prior to the mechanical testing. For extremely fragile samples (i.e. PLCL conditions), the aluminum foil was left on while the sample was glued between slides to provide support during hydration. After hydration, measurements of the sample thickness and width were taken at three locations along the narrow part of the sample as well as the gauge length.

#### 2.6.1. Tensile tests

The exceed model E43 universal test machine (MTS Systems Corporation, Eden Prairie MN) with a 250 N load cell was used for tensile tests. Samples were loaded into the machine and secured between the grips using double-sided tape on the plexiglass slides to prevent slipping. Samples were stretched into slight tension, and the preload was recorded prior to the test. For extremely thin samples, the foil was left on during hydration and broken during this time. Tensile tests were conducted at a speed of 2 mm min^−1^ and samples were stretched until rupture.

Engineering strain and stress were calculated from the data outputs of crosshead displacement (mm) and load (N) recorded by the machine in TWE elite, combined with measurements of the material thickness and width from the region of break. Curves were processed using Excel and then sampled down to 50 samples in MATLAB to standardize data

The linear region of the curve was first determined visually and confirmed by comparison across curves quantitatively. Young’s modulus was calculated using the slope of two points along the linear region selected to be comparable across each condition. Ultimate tensile strength (UTS) was selected as the peak of the data. Strain-at-break was determined by matching the coordinate at the ultimate tensile stress to its strain value.

#### 2.6.2. DMA

DMA Q800 (TA Instruments, New Castle, DE) was used in static tension mode to determine the elastic modulus of the specimens. Each specimen was first subjected to a monotonic strain ramp until rupture. In a separate test, a controlled strain ramp from 1% to 10% was applied at a rate of 1% strain per minute, and the modulus was calculated from the initial linear portion of the stress–strain response.

#### 2.6.3. Suture retention strength

Suture retention strength was evaluated using five scaffold sheets measuring 1 × 2 cm. A 5–0 Deme CRYL^TM^ (DemeTECH) suture was placed 2 mm from the 1 cm edge of each sheet and tied to form a loop. Testing was performed on a DMA Q800 (TA Instruments, New Castle, DE). The knotted suture end was secured in the upper clamp, while the scaffold sheet was fixed in the lower clamp. The suture was pulled at a rate of 5 mm min^−1^ until it tore through the scaffold, in accordance with ISO 7198. The peak load for each sample was recorded in Newtons (N) and averaged, assuming a sheet thickness of 100 μm [21]. The specimen thickness might also affect the measured force, so some authors adopted a thickness-normalized suture retention strength (SRS) definition [22]. All values were normalized to sample thickness to allow direct comparison between groups and the literature.

### 2.7. Hemocompatibility study

The platelet adhesion process was initiated by obtaining platelet-rich plasma (PRP) from pooled human plasma (Innovative Research, Novi, MI). The samples were sterilized for 15 min under UV light and then submerged in the PRP for either 1 or 6 h at 37 °C, while gently shaking. Samples were washed twice in PBS after PRP submersion to wash away unattached platelets, then preserved with glutaraldehyde and sterilized with ethanol before being desiccated and imaged under SEM. The SEM images were quantified by counting the number of adhered platelets per area.

Thrombogenic potential was evaluated by monitoring platelet-poor plasma (PPP) clot formation via absorbance kinetics. PPP was prepared from fresh bovine blood (Lampire Biological Laboratories, Pipersville, PA). Tubular-shaped scaffolds (5 mm diameter) were prepared for each experimental condition, with glass slides and expanded ePTFE (GORE-TEX®, 5 mm) grafts serving as controls. All samples were sterilized under UV light for 15 min and prehydrated in PBS for 90 min before testing.

The assay was conducted according to our previously published protocol [23]. Briefly, hydrated samples were placed directly into a 96-well plate, and PBS was replaced with 50 μl of HEPES-buffered saline, platelet-poor bovine plasma, and 25 mM calcium chloride solution. Absorbance at 405 nm was recorded every 51 s for 1 h using a microplate reader. Clotting onset time (10%) was determined from the absorbance curves and reported along with representative kinetic profiles.

### 2.8. Materials subcutaneous implantation and explant evaluation

Animal experiments were performed according to the IACUC requirements and complied with the NIH’s guidelines for the care and use of laboratory animals.

#### 2.8.1. Surgery

The *in vivo* observation of the groups under investigation was performed by placing the samples under the skin of rats. The surgery was performed on male Sprague Dawley rats at 10–11 months old at the time of the implantation, weighing ∼ 400 grams. The rats were purchased from ENVIGO (Indianapolis, IN). Scaffolds were sterilized prior to implantation by immersion in 70% ethanol for 30 min, followed by exposure to UV light for 15 min. These methods are commonly applied to polymeric biomaterials and have been shown to preserve scaffold bioactivity and minimize alterations to host immune responses [24, 25]. The subcutaneous implant proceeded as follows. The rats were anesthetized with isoflurane (2%) using an induction chamber and vaporizer with the use of a nose cone to maintain anesthesia. Warm lactated ringer solution was administered for rehydration (2.0 ml/100 g Body Weight of Rat.) via subcutaneous injection. The abdominal area was shaved and disinfected using 80% ethanol, followed by provo-Iodine. A midline abdominal incision was made, separating the skin to expose the surface of the abdominal wall muscle layer (2.5-inch midline incision). Samples were sterilized with 70% ethanol for 15 min and rinsed three times with sterile PBS, they were preserved at 4 °C and soaked in PBS for 1 h prior to implantation. Between the abdominal wall and the hidden layer, 6–8 biomaterial samples with a PTFE O-ring frame (5/16 inner diameter, RT Dygert, Burnsville, MN) were inserted subcutaneously using a small hemostat to open a small pocket. This was repeated up to 4 times along each side of the 2.5-inch midline incision for a total of 8 samples. Once samples were placed in each pocket, the hidden layer was closed using 9 mm wound clips. A 50/50 mix of lidocaine 1%–2% mixed with 0.5% bupivacaine was applied along the midline incision as a local analgesic, as well as Meloxicam SRTM by subcutaneous injection on the back for a slow-release analgesic. A total of 9 rats were used (*n* = 3 per time point), with up to 8 subcutaneous pockets created per rat. For each condition at each time point, four constructs were implanted and evaluated.

#### 2.8.2. Explanation and sample preservation

The samples were retrieved at time points 1, 4, and 16 weeks, at which time the animals were sacrificed. Implantation time points were selected to capture distinct phases of host response and scaffold performance. The 1-week timepoint reflects the acute inflammatory phase, while 4 weeks corresponds to early remodeling and tissue integration [26]. The 16-week timepoint was chosen to represent a mid-term stage, when electrospun PCL scaffolds begin to show measurable reductions in molecular weight and mechanical integrity but before bulk mass loss occurs, as reported in prior studies [27]. Subcutaneous implants were explanted with the same procedure at the specific time point, with efforts made to minimize the collection of extra tissue surrounding the sample. Each retrieved specimen was cut in half: one part was fixed with 10% formalin and embedded in paraffin for cutting, histological staining, and imaging. The other half was preserved in the optimal cutting temperature compound (OCT) and then rapidly frozen in liquid nitrogen for immunological staining. Slides were cut from the paraffin-embedded samples at 5-micrometer thickness.

#### 2.8.3. Histology

Hematoxylin and eosin (H&E) stain from cancer diagnostics (Durham, NC) was performed by the histology core. H&E-stained slides were observed with light microscopy (Nikon Eclipse Ti), and the images taken were used for qualitative analysis of material behavior, implemented by quantitative results via Fiji ImageJ on the thickness variation over time of the different conditions. For quantitative analyses, three constructs per condition that met quality criteria—defined by intact explants after histological processing and adequate staining quality—were included. From each construct, five representative regions were analyzed and averaged, resulting in *n* = 15 data points for quantitative parameters such as thickness.

#### 2.8.4. Immunofluorescence

Immunofluorescence analysis was performed using CD68 and CD206 as markers of macrophages and the M2 phenotype, respectively. Macrophages were identified with anti-CD68 (sc-20 060, Santa Cruz Biotechnology, Dallas, TX), while M2 macrophages were detected with anti-CD206 (abx140463, Abbexa LLC, Houston, TX). Slides were rehydrated and probed overnight at 4 °C at a dilution of 1:100. Coverslips were applied with Vectasheild mounting medium with DAPI for counter-stain (Vector Laboratories, Burlingame, CA) and imaged with a fluorescent microscope (Nikon, Instruments Inc., Melville, NY).

#### 2.8.5. Multiphoton and fluorescence microscopy

For multiphoton microscopy, paraffin-embedded, unstained tissue sections were rehydrated before observation. The deparaffinization process involved sequential soaking of the slides: first in Histo-Clear for 10 min, followed by a 1:1 mixture of Histo-Clear and ethanol (EtOH), then in serial dilution of ethanol (100%, 95%, 70%, 50%), and finally in deionized (DI) water. Each step, except the initial treatment, was performed for 5 min. Samples were preserved in DI water until imaging.

Multiphoton imaging techniques, including second harmonic generation (SHG) and two-photon excitation fluorescence (TPEF), were employed to quantify collagen and elastin, respectively. Observations were conducted using a GEMINI—Olympus FVMPE-RS twin-laser multiphoton microscope (Olympus, Japan). Excitation was provided by a femtosecond-pulsed laser system tuned to an 860 nm wavelength (Spectra-Physics MaiTai Wideband mode-locked Ti:Sapphire laser system). The response signal was split at 455 nm using a dichroic mirror (AT455 DC, Chroma Technology, Bellows Falls, VT). Collagen SHG (400–455 nm) and elastin TPEF (460–610 nm) signals were simultaneously captured using a non-descanned direct detection system (Bio-Rad, Boulder, CO).

Multiphoton images highlighted the presence of native tissue (collagen and elastin) in the different conditions and time points. The images taken were used as an indicator to distinguish synthetic material from native tissue, allowing us to better evaluate cell infiltration in our samples. Given the location of implantation of the sample, no further efforts were made to distinguish elastin and collagen content.

After multiphoton observation, the same slides were used for further observation. Fluorescent images revealed each condition’s native tissue integration and degradation properties. In particular, the samples were stained with maleimide to localize the synthetic material and DAPI for cell nuclei. Maleimides are electrophilic compounds that react with thiols to form a stable thioether linkage [28], and the availability of free thiols was quickly checked by calculating the excess moles of the crosslinker (PEG dithiol). The combination of these two stainings allowed us to visualize the cell infiltration in the synthetic material.

A maleimide stain solution of 100 µM was prepared using maleimide from Vector Laboratories in PBS. Each sample was covered with 100 µl of maleimide stain solution overnight and stored at −20 °C. Then, samples were washed briefly in PBS. DAPI stain (Invitrogen, Waltham, MA) was used according to the instructions. Samples were stained for 30 min with DAPI and then rinsed in PBS. A small drop of Vectashield mounting medium for fluorescence with DAPI (Vector Laboratories) was added over the top of each sample, and cover glass was placed over the top, avoiding the creation of bubbles. Slides were stored at −20 °C before imaging.

Images were taken on a Nikon Eclipse Ti microscope in SPOT basic. For compatibility, the maleimide (green) and DAPI (blue) images were taken in the same locations for all samples, approximately in similar regions imaged by multiphoton.

For quantitative analysis maleimide and DAPI images were taken in three different spots for each sample, and the material area must be visible in these images. Three slides from each condition were imaged, for an *n* = 9 for each group at each time point.

Cell counting was done via MATLAB script. Channels for both maleimide and DAPI were processed separately. A binary mask was applied to DAPI images with a minimum cell size of 60 pixels, and images were cleaned using image erosion and opening. Cells were counted from the masked image. maleimide images were quantified using the number of green pixels in the image, but the same binary mask method was adopted.

Cell density counting was performed by overlaying a polygon around the identified material area and counting the cells and green pixels within the polygon. Cell numbers were normalized against the total area analyzed in the mask to ensure comparability between different material areas.

### 2.9. Data analysis and statistics

All quantitative results were exported to an excel file and analyzed in Origin Pro. Statistical analyses on morphology characterization (pore and fiber diameter), thickness variation, mechanical properties (Young’s Modulus, Strain at Break, and UTS), and cell counting results were performed in origin pro. A one-way analysis of variance (ANOVA), followed by a post hoc Bonferroni test, was performed. The error bars represent standard deviations, which capture sample-to-sample variability.

## 3. Results and discussion

### 3.1. Scaffold morphology (SEM/TEM)

**Figure 2.**
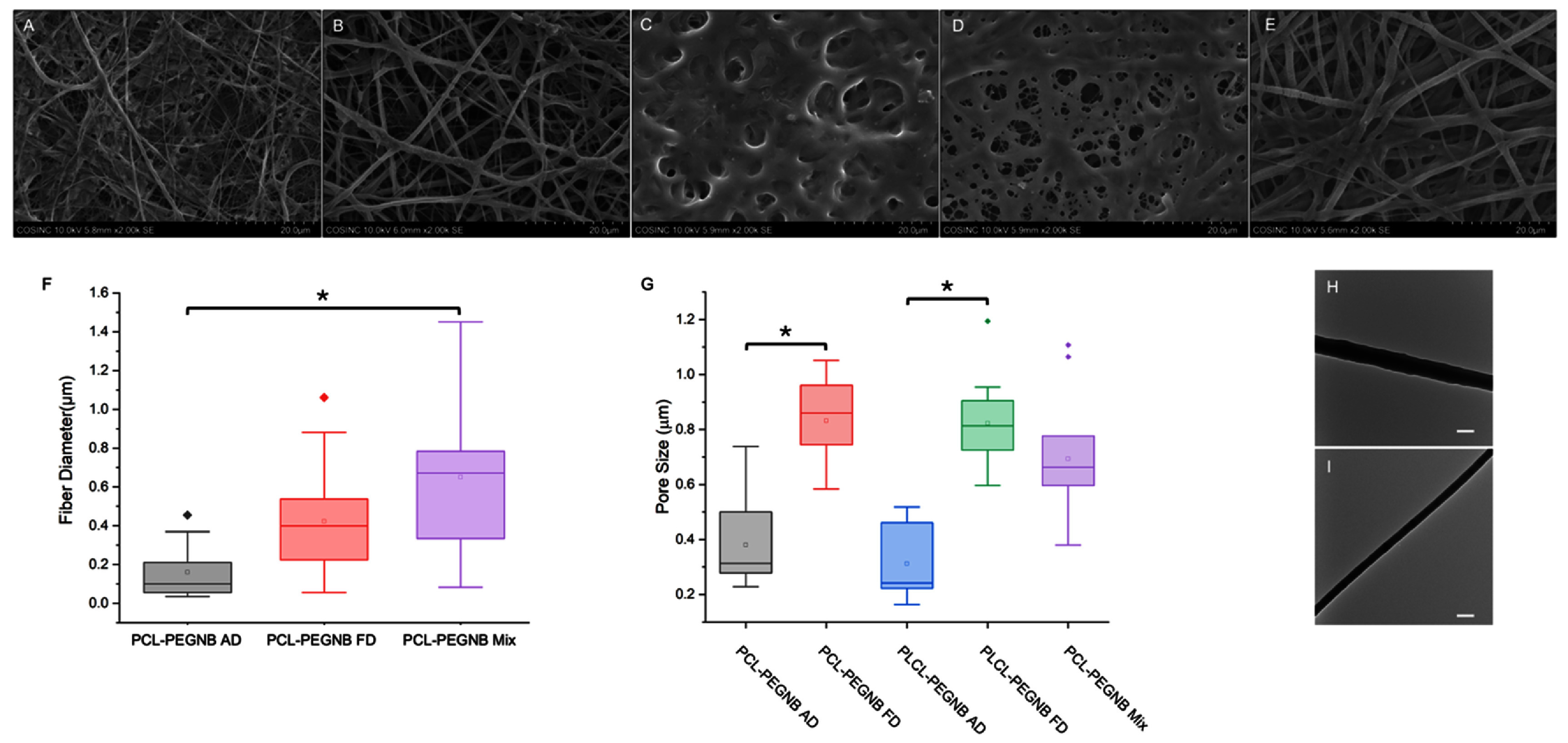
Representative electron microscopy images and quantitative analyses of fibrous scaffolds. (A–E) SEM images of hydrated coaxial fibers made of (A) PCL-PEGNB AD, (B) PCL-PEGNB FD, (C) PLCL-PEGNB AD, (D) PLCL-PEGNB FD, and hydrated PCL-PEGNB Mix (E). (F) Fiber diameter of hydrated PCL-PEGNB groups. (G) Pore size of all groups. ‘*’ statistical difference *p* <0.05. (H and I) TEM images of coaxial fiber PCL-PEGNB AD (H) and PCL-PEGNB Mix (I). Scale bar = 500 nm.

Electron microscopy was employed to examine the morphology of electrospun fiber mats. Figure 2 presents SEM images of the five groups under investigation. Structural differences were observed between PCL-PEGNB groups (figures 2(A), (B), and (E)) and PLCL-PEGNB groups (figures 2(C) and (D)): the PLCL copolymer caused the fiber network to collapse into a mesh-like configuration after hydration. Additionally, the freeze-drying post-processing method (figures 2(B) and (D)) was found to increase porosity compared to AD (figures 2(A) and (C)).

The mean fiber diameters of the three PCL-PEGNB groups range from 0.1 to 0.7 µm (figure 2(F): AD = 0.16 µm, FD = 0.42 µm, Mix = 0.65 µm), with a significant difference observed between AD and Mix groups. The larger fiber diameter in the PCL-PEGNB Mix condition can be attributed to the heterogeneous distribution of the hydrophobic core and hydrogel sheath components, which results in greater swelling during the hydration process.

Figure 2(G) highlights significant differences in pore size between PCL-PEGNB AD and FD (0.38 µm vs. 0.83 µm, respectively), and between PLCL-PEGNB AD and FD (0.31 µm vs. 0.82 µm, respectively), demonstrating the impact of the freeze-drying process on scaffold structure. The increase in pore size and overall porosity resulting from freeze-drying is beneficial for enhancing cell infiltration and promoting tissue regeneration [29]. Although the average pore sizes measured by SEM appear smaller than typical vascular cell dimensions, SEM captures only surface features. Electrospun scaffolds are multilayered fibrous networks, where overlapping fibers across multiple planes likely provides routes for cell penetration. More importantly, freeze-drying effectively increased porosity in both PCL- and PLCL-based scaffolds, underscoring its role in improving scaffold architecture for cell recruitment.

TEM images provide a detailed visualization of the single fiber structure, highlighting differences between coaxial (figure 2(H)) and mixed (I) electrospun fibers. The sheath and core fiber configuration are particularly evident in the PCL-PEGNB coaxial group. In the PCL-PEGNB Mix group, no gradient along the fiber is observed due to the homogenized blend of the two solutions. In contrast, fiber organization is less distinct in the PLCL-PEGNB coaxial group (supplementary figure S1). Some fibers exhibit ruptures in the sheath, exposing the core polymer.

### 3.2. XPS

An XPS survey showed C 1s and O 1s, as expected, and Si 2p, S 2p, and N 1s. The strong silicon signal was due to the glass substrate; the gold coating was adopted to mask it. The results from the high-resolution scan on C1s are reported in Supplementary Materials. The coaxial fibers, which we anticipated to look just like PEG-NB, therefore only C–O bonds, showed strong C–C. The unexpected results could be justified by the capillary rupture of the sheath prior to photocrosslinking, as observed in TEM images (supplementary figure S2). This phenomenon led to the exposure of the core solution; therefore, the instrument detected the strong C–C bonds characteristic of PCL and PLCL.

### 3.3. Mechanical characterization: tensile test and DMA

**Figure 3.**
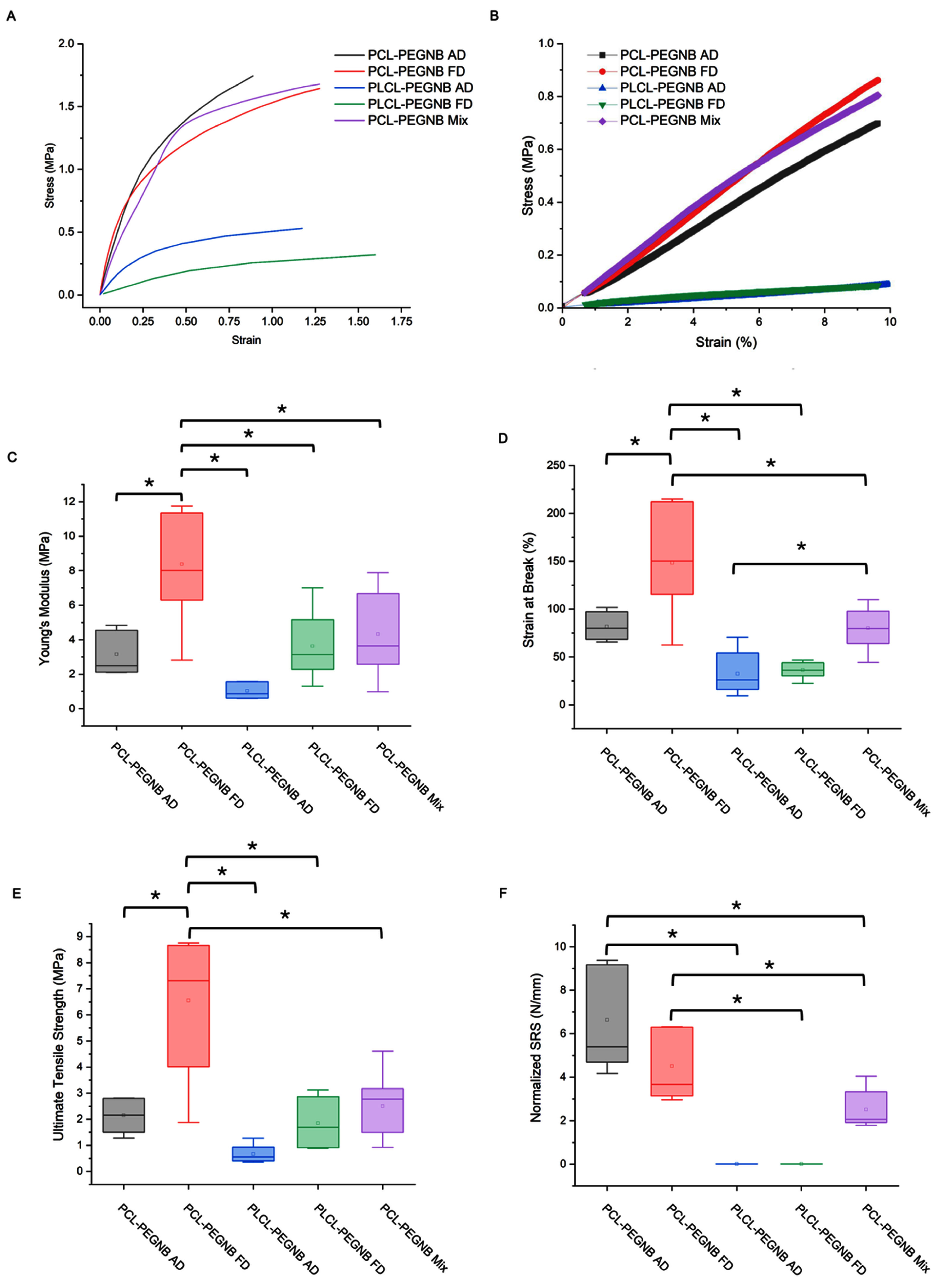
Representative stress–strain curves and quantitative tensile properties of PCL-PEGNB and PLCL-PEGNB fibrous scaffolds, using DMA (A, B, and F) and tensile test machines (C, D, and E). (A-B) Curves showing stress–strain relationship until rupture (A) and in the elastic range of 1%–10% (B). (C) Young’s modulus, (D) strain at break, (E) ultimate tensile stress, and (F) normalized suture retention strength. ‘*’ statistical difference *p*< 0.05.

To evaluate the graft’s mechanical properties, we quantified the Young’s modulus, strain at break, and ultimate tensile strength from tensile test curves (supplementary figure S3). Figure 3(C) shows the Young’s modulus for all groups. Notably, the Young’s modulus of PCL-PEGNB FD was significantly higher than that of all other groups (*p* < 0.05), with an average value of 8.83 MPa. The freeze-drying process substantially increased the modulus compared to the PCL-PEGNB AD condition, which had an average modulus of 3.15 MPa. This enhancement brought the modulus closer to native vessel properties, such as the circumferential and longitudinal moduli of the left internal mammary artery, which are approximately 8 MPa and 9–12 MPa, respectively [30]. This improvement is attributed to the increased rigidity of the hydrogel sheath induced by the freeze-drying process [31]. Given that vascular grafts undergo repetitive low-strain expansion and contraction due to the pulsatile nature of blood flow, closely matching the Young’s modulus of the native vessel is critical for optimal graft design.

Strain-at-break measurements (figure 3(D)) revealed that PCL-PEGNB FD had the highest strain at break, averaging 148%, significantly surpassing all other groups. This value closely matches the circumferential strain of the internal mammary artery, reported as 134% [30]. Intriguingly, the PLCL-PEGNB AD condition did not exhibit an increased strain at break compared to PCL-PEGNB AD; instead, it showed a slight decrease (*p* = 0.0527). This outcome seemingly contradicts previous literatures, which report significantly higher strain values for PLCL compared to PCL [32]. This discrepancy is attributed to fiber collapse within the PLCL-PEGNB material. Unlike the robust fibrous structure observed in PCL-PEGNB coaxial designs, the PLCL-PEGNB conditions lacked sufficient structural integrity, preventing effective mechanical load distribution across the fiber network. Consequently, the plastic deformation of the hydrogel might play a predominant role, leading to reduced strain-at-break values and causing samples to fracture more readily than their PCL-PEGNB counterparts, diverging from the expected strain properties of PLCL-PEGNB.

In ultimate tensile stress measurements (figure 3(E)), PCL-PEGNB FD again outperformed all other groups, achieving a significantly higher average value of 6.55 MPa, surpassing the circumferential yield stress of the internal mammary artery (4.1 MPa) [30]. Although the yield stress values of the other groups were not significantly different, it is noteworthy that the AD and Mix conditions of PCL-PEGNB exhibited similar averages (2.15 MPa and 2.5 MPa, respectively), both exceeding those of the PLCL-PEGNB conditions (AD: 0.66 MPa, FD: 1.84 MPa).

**Figure 4.**
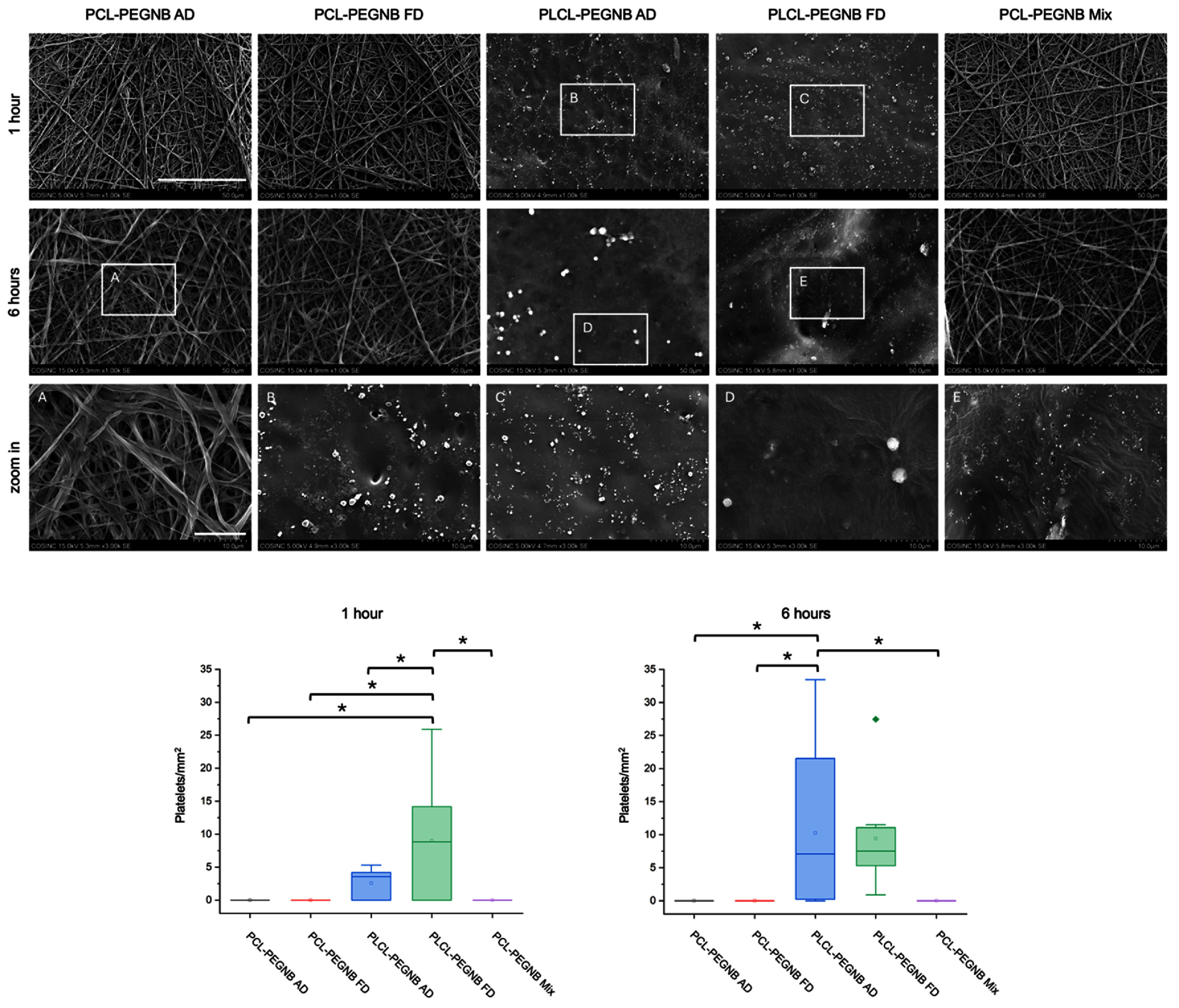
Representative SEM images (Top panel) and quantitative analyses (bottom panel) of platelet adhesion study. Scale bar = 50 μm, zoom-in images scale bar = 10 μm. ‘*’ statistical difference *p* <0.05.

The stress-strain curves taken with the DMA Q800 are presented in figures 4(A) and (B). A strain range of 1% to 10% was selected to evaluate the elastic region, which is critical for vascular graft application to maintain elastic functionality under pulsatile flow without entering the plastic deformation region. Moduli obtained from DMA (supplementary figure S4) and tensile test machine (figure 3(C)) were compared, showing similar ranges across all groups under static tensile conditions. The only notable difference was observed between PCL-PEGNB and PLCL-PEGNB. This finding confirms that at 1%–10% strain, the scaffolds remain in their elastic regime, where properties are primarily governed by the core polymers, and post-processing techniques have minimal impact on the scaffolds at these low strain levels.

Suture retention testing revealed marked differences among scaffold groups. Suture retention strength data were collected (supplementary figure S4), and the normalized SRS data are reported in figure 3(F). PLCL scaffolds were extremely fragile, with sutures tearing through the material at the initial preload of 0.01 N, preventing reliable measurement. Among the PCL-based groups, PCL-PEGNB Mix exhibited the lowest retention strength (2.5 ± 0.3 N), consistent with its inferior performance in uniaxial tensile testing. PCL-PEGNB FD scaffolds withstood higher loads (4.7 ± 0.5 N), while PCL-PEGNB AD demonstrated the greatest resistance (6.3 ± 0.8 N).

Although PCL-PEGNB FD showed higher tensile modulus and UTS in uniaxial testing, PCL-PEGNB AD exhibited higher suture retention strength. This apparent discrepancy reflects the fact that suture retention is governed by localized mechanics around the suture site, including fiber engagement, inter-fiber friction, pull-out, and local densification, rather than the global axial strength of the scaffold. The smaller fiber diameter and reduced porosity in PCL-PEGNB AD increase the number of fibers that interact with the suture and promote frictional interlock and energy dissipation via fiber pull-out. These features yield greater resistance to suture pull-out despite lower bulk UTS. Conversely, the stiffer PCL-PEGNB FD scaffold tends to concentrate stresses and fail more abruptly at the puncture site.

In summary, mechanical testing reveals that PCL-PEGNB FD closely replicates the mechanical properties of native vessels in terms of Young’s modulus and strain at break. The freeze-drying process enhances the stiffness of PCL-PEGNB scaffolds and significantly increases their ultimate tensile strength. Furthermore, the fibrous structure achieved through coaxial electrospinning of PCL-PEGNB offers superior rigidity and strain performance after hydration, compared to the collapsed structure observed in PLCL-PEGNB scaffolds. Such collapsed structure of PLCL resulted in much lower strain than previously reported [33, 34].

### 3.4. Hemocompatibility

#### 3.4.1. Platelet adhesion study

A PRP adhesion study was conducted to assess the hemocompatibility of the scaffolds, with results shown in figure 4. Due to differences in sample morphology, distinct platelet adhesion behaviors were observed between PCL-PEGNB and PLCL-PEGNB groups. The fibrous structure of the PCL groups effectively prevented platelet adhesion at both 1 and 6 h. In contrast, PLCL initiated platelet adhesion within 1 h, attributed to its smoother surfaces. No significant differences in platelet adhesion were observed within the PCL-PEGNB groups. Quantitative results are reported in figure 4. After one hour, average platelet adhesion was quantified at 2.5 platelets/mm^2^ and 9.0 platelets/mm^2^ for PLCL-PEGNB AD and FD, respectively, while no platelets were detected on any PCL-PEGNB groups. At 6 h, the average adhesion increased to 10.2 platelets/mm^2^ and 9.4 platelets/mm^2^ for PLCL-PEGNB AD and FD, respectively, with PCL-PEGNB groups continuing to show no platelet adhesion. The smoother surface characteristic of the PLCL-PEGNB collapsed structure possibly facilitates platelet adhesion, resulting in a higher platelet count and increased risk of thrombosis formation. Notably, at 6 h, PLCL-PEGNB AD exhibited platelets of larger apparent size compared to the 1 h condition, suggesting the onset of platelet aggregation, whereas in PLCL-PEGNB FD, platelets were present at both time points but remained similar in size, indicating adhesion without significant aggregation.

The observed reduced platelet adhesion can likely be attributed to two complementary factors. First, surface architecture influences thrombogenicity by modulating protein adsorption, local shear interactions, and cell attachment; smoother or more uniform fiber structures may present fewer sites for platelet activation [35]. Second, incorporation of PEG-NB is expected to impart anti-fouling properties due to its hydrophilicity and steric hindrance effects, which limit nonspecific protein adsorption and subsequent platelet binding [36]. The combination of structural effects and PEG-NB chemistry, therefore, provides a plausible mechanism for the reduced thrombogenic potential observed in selected graft designs.

PRP studies revealed that PLCL grafts exhibited the highest degree of platelet adhesion, consistent with their collapsed microstructure, which likely provided more surface area for platelet attachment. In contrast, PPP clotting assays (supplementary figure S5) demonstrated that PLCL had the longest time to fibrin clot formation, suggesting reduced fibrin generation despite the enhanced platelet adhesion observed in PRP. These complementary findings highlight the distinct behaviors of PLCL under platelet-rich versus platelet-poor plasma conditions. Notably, all experimental groups displayed longer coagulation times compared with commercial ePTFE controls.

### 3.5. Materials subcutaneous implantation and explant evaluation

All animals survived the procedure without evidence of sepsis or infection, and there were no different macroscopic findings between groups. (supplementary figure S6)

#### 3.5.1. Histology and quantitative analysis

**Figure 5.**
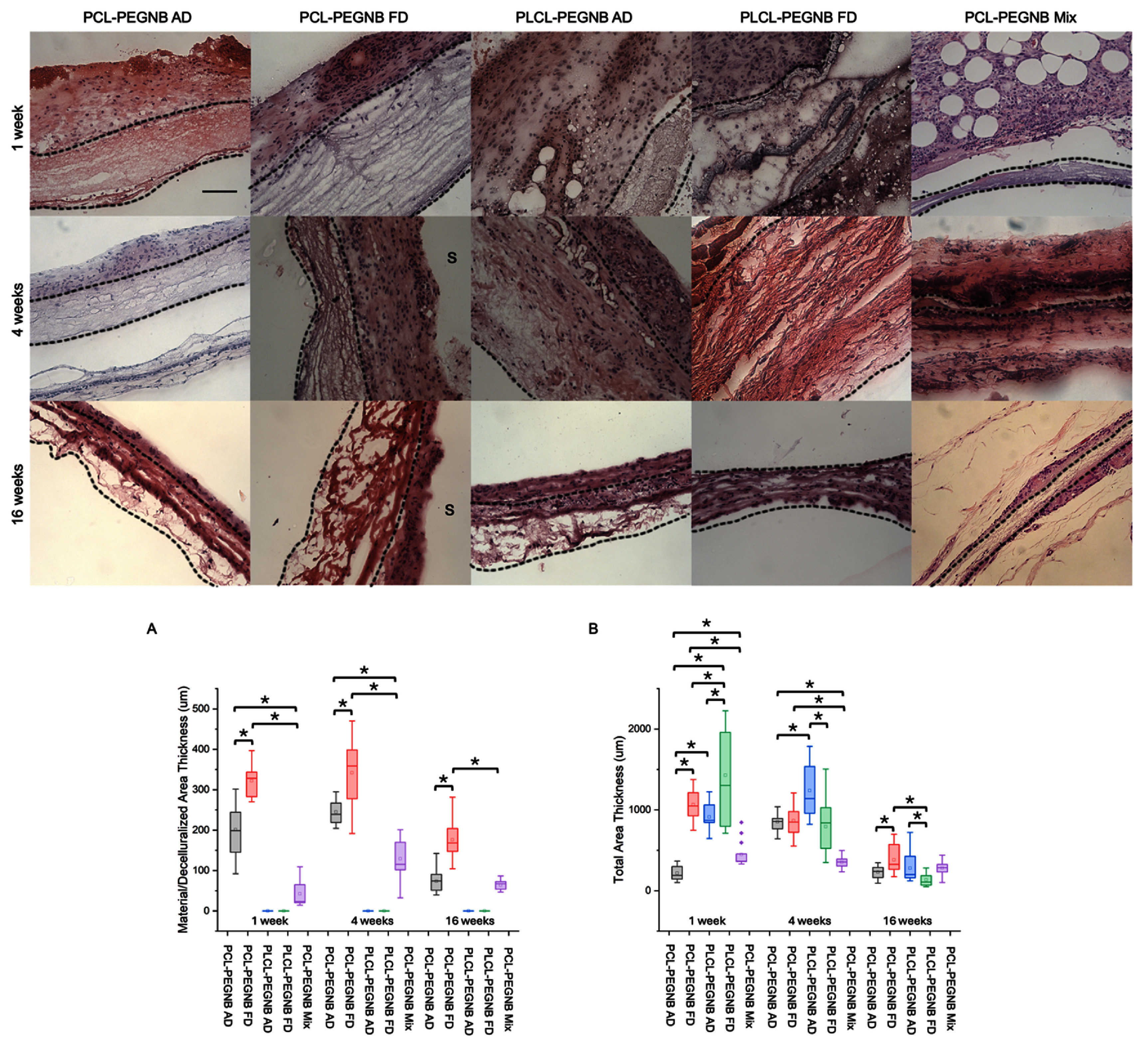
Histological analysis showing cell infiltration and tissue remodeling over 1, 4, and 16 weeks. The top panel shows representative H&E stain images of infiltrated cells in the explanted grafts. Black dotted lines indicate the area of implant materials. Scale bar = 100 μm. All images were taken at 10x magnification. Images are oriented skin top and the dermis bottom, unless specified with an ‘s’ indicating the skin side. Bottom panel (A) shows material/decellularized area thickness, and (B) shows total area thickness of explanted grafts. ‘*’ statistical difference *p* < 0.05.

Images from H&E-stained slides, presented in figure 5, reveal differences in the extent and pattern of cellular infiltration and extracellular matrix deposition across groups. The synthetic material was more prominent in the PCL-PEGNB groups compared to the PLCL-PEGNB groups. Both AD and FD of PCL-PEGNB exhibited a gradual decrease in thickness over time, indicating slow material integration with native tissue. A notable difference between PCL-PEGNB AD and FD was observed: the freeze-drying process resulted in a more porous structure, not only on the surface (as shown in SEM images) but also throughout the thickness of the specimen.

In contrast, the PLCL-PEGNB groups integrated with animal tissue immediately after implantation, making it difficult to distinguish the synthetic material from the extracellular matrix at 1 week. Additionally, both AD and FD of PLCL-PEGNB showed signs of degradation, with fragmentation of the graft and a reduced thickness of the combined material and tissue area.

PCL-PEGNB Mix displayed an intermediate behavior: while the material region was easier to identify than in the PLCL-PEGNB groups, it was not as distinct from the tissue as in the PCL-PEGNB coaxial groups.

The thicknesses of the entire implant and the decellularized region (material only) were measured using ImageJ, with results presented in figure 5. In the PLCL-PEGNB groups, cells infiltrated the entirety of the samples, resulting in a decellularized region thickness of nearly zero. Among all groups, the PCL-PEGNB FD exhibited the thickest decellularized region at each time point (figure 5(A)), indicating that the freeze-drying process significantly increased the total material thickness. The decellularized region remained similar in the first 4 weeks for all PCL-PEGNB groups.

Regarding total thickness measurements (figure 5(B)), at 1 week, PLCL-PEGNB FD exhibited the largest thickness among all five groups, although with the highest variation. PCL-PEGNB FD showed a significant increase in thickness compared to the other PCL-PEGNB conditions. By 4 weeks, PCL-PEGNB AD and FD demonstrated similar thickness values, both significantly greater than the Mix group. Interestingly, PLCL-PEGNB AD exhibited significantly larger thickness than PLCL-PEGNB FD at this time point. These findings suggest that the structural effects of freeze-drying, which enhance porosity and thickness, are counterbalanced by increased cellular infiltration, resulting in cellular traction and tissue compaction.

At 16 weeks, the thickness values across groups were generally lower than at 1 and 4 weeks, indicating significant tissue remodeling and cellular compaction. A reduction in material thickness was also observed, consistent with expected degradation (lower values were also reported for the decellularized/material region at 16 weeks). Notably, a significant difference in thickness was observed between PCL-PEGNB AD and FD at 16 weeks, while PLCL-PEGNB AD showed significantly larger total thickness compared to PLCL-PEGNB FD. These findings highlight the complex interplay of factors such as cellular traction, material degradation, and extracellular matrix production, all of which influence thickness during later stages of *in situ* tissue regeneration.

#### 3.5.2. Immunofluorescence study of macrophage activity

An evaluation of macrophages using CD68 and CD206 antibodies was conducted, revealing very few positive cells across all samples and time points (supplementary figure S7). These findings suggest a minimal level of inflammation, especially when compared to other studies involving subcutaneous grafts [37]. This indicated a minimal inflammation level compared to other studies with subcutaneous grafts. One possible reason could be associated with the use of a PTFE ring as a frame, which, according to previous studies [38], limits the activity of macrophages on the samples.

Evaluation of macrophage polarization revealed the presence of both M1- and M2-like populations within the scaffold region. Polarization toward an M2-like phenotype is generally linked to constructive remodeling and vascular integration, whereas M1 macrophages reflect a pro-inflammatory response [39]. This balance is particularly relevant for AV graft applications, where early immune responses are known to influence long-term patency outcomes [40].

#### 3.5.3. Multiphoton, fluorescence, and cell counting

**Figure 6.**
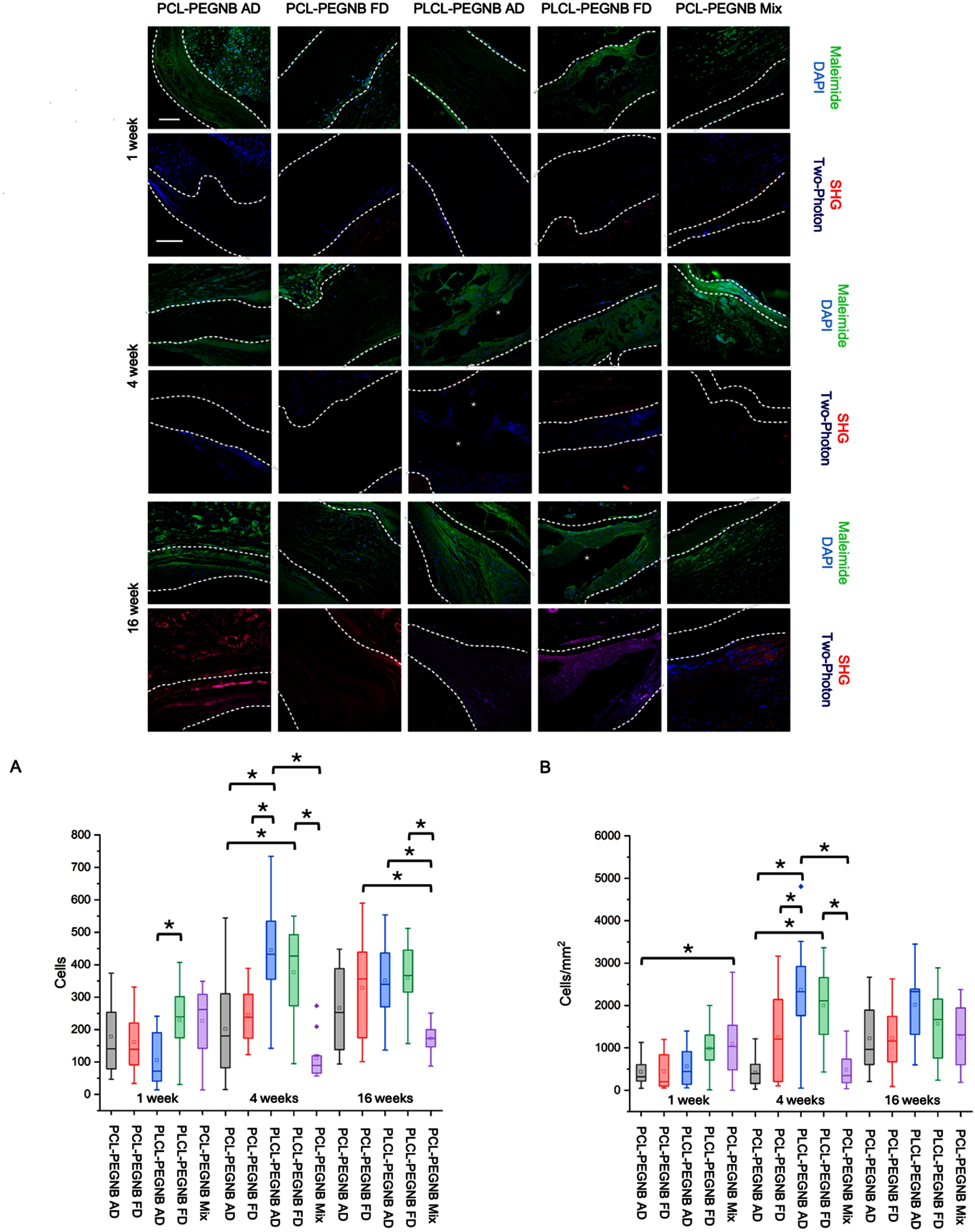
Fluorescent and multi-photon images showing cell infiltration, ECM production, and tissue remodeling over 1, 4 and 16 weeks. (Top panel) For each time point, top row shows DAPI and maleimide staining of cell nuclei and thiolated material, respectively and bottom row shows two-photon images showing collagen deposition (red, from SHG imaging) and elastin (blue, from TPE imaging). White dotted lines show implant area and ‘*’ shows delaminated areas caused by histological sectioning. Scale bar = 100 μm. Bottom panel (A) shows the total number of cells for entire implant and (B) shows cell density in the material area, highlighted between the white dotted lines in the top panel images. ‘*’ statistical difference *p* <0.05.

To investigate changes in the materials, samples stained with maleimide and DAPI were visualized using fluorescence microscopy. The production of extracellular matrix by cells infiltrating the grafts was assessed using SHG and TPEF to visualize collagen and elastin, respectively (figure 6). Native tissue integration was quantified by counting the total number of cells (figure 6(A)) within the full image (encompassing both native tissue and material) and by measuring cell density specifically within the material area (figure 6(B)).

At 1 week, the PCL-PEGNB groups displayed a distinct boundary between the native tissue and the material area, with cells localized only along the outer edges (figure 6). This cell infiltration pattern at the one-week time point aligns with observations from the histological images. The PLCL-PEGNB FD condition demonstrated greater tissue integration compared to PLCL-PEGNB AD in terms of total cell count (figure 6(A)), while the PCL-PEGNB Mix condition showed improved cell penetration (cell density within the material area, figure 6(B)) compared to PCL-PEGNB AD. Although these data reflect overall host cell infiltration, they indicate early scaffold–tissue interactions that may represent the initial stages of vascular-specific remodeling, which will be further clarified in future lineage-specific analyses.

These differences can be attributed to the physical properties of the materials. The smoother and softer surface of the collapsed PLCL-PEGNB structure promoted better tissue interaction and cell adhesion compared to the rigid fibrous structure of the PCL-PEGNB conditions. The increased cell density in PCL-PEGNB Mix is likely due to the heterogeneous distribution of PCL and PEG-NB polymers, which facilitated cell infiltration compared to the more uniform coaxial structure of PCL-PEGNB AD.

At 4 weeks, the most significant differences among the groups were observed compared to the 1-week and 16-week time points. No differences in cell density or total cell count were detected between the PLCL-PEGNB AD and FD conditions. Notably, the PLCL-PEGNB AD condition exhibited significantly higher cell density and total cell count than all three PCL + PEG-NB conditions. This result can be attributed to the surface morphology and degradation behavior of the PLCL material. The enhanced native tissue integration observed in the PLCL-PEGNB groups compared to PCL-PEGNB conditions aligns with the findings from the histological analysis, particularly the total thickness measurements (figure 6). Such robust infiltration at 4 weeks may represent a transition from nonspecific host cell recruitment toward tissue remodeling events that are prerequisite for endothelial and smooth muscle regeneration.

At 16 weeks (figure 6), the PCL-PEGNB Mix condition exhibited a significantly lower cell count compared to all groups except for PCL-PEGNB AD. While no significant differences in cell density were observed quantitatively across all materials, the images revealed that the PCL-PEGNB Mix condition significantly restricted cell infiltration to the surface. This limitation can be attributed to the hydrophobic nature of PCL, which is directly exposed to the tissue in the mixed group, thereby hindering cell adhesion [41]. The low adhesion between materials of distinct properties in the Mix condition may result in undefined material defects in heterogeneous scaffolds. This could also explain the difficulty in handling the PLCL-PEGNB Mix condition.

Overall, cell density exhibited a general tendency to increase across time points, with peak increases occurring at different intervals depending on the core polymer (supplementary figure S8). PLCL-PEGNB AD and FD showed a significant increase in cell density between 1 and 4 weeks, but no significant change beyond 4 weeks. This is likely due to the faster cell penetration observed in PLCL-PEGNB compared to the PCL-PEGNB groups. In contrast, PCL-PEGNB AD demonstrated a significant increase in cell density between 4 and 16 weeks, with no substantial change during the earlier time points, suggesting a longer timeframe is required for sufficient tissue integration. PCL Mix, however, did not show any significant increase in cell density across the observed time points.

## 4. Discussion

The present study systematically evaluated how polymer selection, scaffold architecture, and post-processing influence the performance of coaxially electrospun PEG-NB hybrid scaffolds in the context of vascular grafts. By comparing PCL–PEGNB and PLCL–PEGNB scaffolds, assessing post-processing effects, and applying advanced imaging to study host responses, this study offers new insights into electrospun vascular graft design with relevance to AV shunt applications.

In this study, PTFE rings served only as mechanical supports during implantation rather than experimental controls, as our true control condition was the PCL + PEG-NB AD scaffold, consistent with our prior work [17]. Supplementary figure S6 confirms that PTFE rings did not retain tissue and were readily removed, indicating that integration was driven by scaffold properties themselves. Differences in platelet adhesion across scaffold groups further highlight how surface topography and polymer chemistry influence thrombogenic potential, with PEG-NB incorporation likely contributing anti-fouling properties that reduce protein adsorption and platelet attachment, consistent with prior reports of PEG-based biomaterials mitigating thrombus formation [36]. Our platelet assays incorporated shaking-induced shear stress to approximate physiological conditions, thereby reducing nonspecific sedimentation and providing a more relevant assessment than static incubation [42, 43]. These preliminary findings suggest improved thromboresistance compared to conventional synthetic grafts, but validation under physiological flow and extended implantation will be critical.

Subcutaneous implantation, while widely used as a first step to assess biocompatibility, inflammation, and early integration, does not fully replicate vascular remodeling due to the absence of hemodynamic forces, immune factors, and extracellular matrix context unique to the arterial environment [44, 45]. Indeed, arterial implantation studies of electrospun grafts in rodent models have demonstrated more physiologically relevant insights into remodeling and patency [46]. Nevertheless, subcutaneous models remain invaluable for screening scaffold performance. Our timepoints of 1, 4, and 16 weeks were selected to capture the temporal sequence from acute inflammation (1 week) through early remodeling (4 weeks) and mid-term degradation and host remodeling (16 weeks), aligning with established degradation profiles of PCL-based scaffolds [47, 48].

To further distinguish scaffold material from tissue, maleimide staining was applied, exploiting maleimide-thiol binding to PEG-NB structures. Electrospun samples served as positive controls and native vessels as negative controls, the latter confirming elastin autofluorescence and cysteine contributions that can complicate interpretation, especially at later degradation stages, though structural and intensity differences still enabled differentiation. Multiphoton imaging complemented histological evaluation by providing label-free, high-resolution visualization of collagen and elastin fibers, consistent with Picrosirius Red staining but with superior structural detail [49, 50]. This technique additionally offered optical sectioning of thick tissues, enabling a three-dimensional perspective of extracellular matrix organization. Importantly, SHG imaging detected fibrillar collagens, primarily type I with contributions from type III, and given that scaffolds lacked fibrillar collagen pre-implantation, the signal can be attributed to de novo deposition [51]. Although elastin organization is limited in early stages of small-caliber graft remodeling, its evaluation is critical as it underpins long-term compliance and vascular functionality, underscoring the value of multiphoton imaging in scaffold–host interaction studies [52].

These findings add to the growing body of literature on electrospun vascular grafts, where pore size, fiber alignment, and polymer chemistry are known to influence cell infiltration, thrombogenicity, and remodeling outcomes [18]. PCL and its copolymers remain the most widely studied synthetic materials for small-diameter vascular grafts due to their processability, biocompatibility, and tunable degradation, though their slow resorption and risk of neointimal hyperplasia remain challenges [15]. PEG-based functionalization strategies, as employed here, have been reported to enhance hydrophilicity and reduce fouling, improving hemocompatibility and tissue integration compared to unmodified PCL [36].

In the context of clinical translation, small-caliber grafts are needed for both cardiovascular indications (e.g. coronary and peripheral bypass) and AV shunts for hemodialysis access, two applications that share similar patency challenges but differ in mechanical loading and host responses. AV shunts, in particular, remain a critical unmet need, with PTFE conduits still serving as the clinical standard despite high failure rates [53]. The structural reinforcement and biofunctionalization strategies explored here align with ongoing efforts to engineer more durable AV grafts while also being broadly relevant to peripheral and coronary bypass grafts. Thus, our findings not only provide insight into scaffold design parameters but also underscore the translational relevance of coaxially electrospun PCL-PEGNB grafts across multiple small-diameter vascular applications.

Finally, as our current work is limited to subcutaneous implantation models, scalability considerations remain a longer-term goal. The next critical step will be the fabrication and evaluation of tubular grafts, after which strategies for translating coaxial electrospinning from laboratory setups to industrial manufacturing can be systematically addressed.

## 5. Conclusions and future work

This study demonstrates how polymer choice, scaffold architecture, and post-processing collectively influence the performance of electrospun PEG-NB hybrid scaffolds intended for vascular applications. Direct comparison of coaxial versus blended formulations highlighted the importance of fiber organization, with coaxial scaffolds enabling more controlled tuning of mechanics and host interactions. Within these designs, PCL–PEGNB provided mechanical robustness, while PLCL–PEGNB supported faster cellular infiltration and remodeling, suggesting complementary roles that could be harnessed in future multilayer or composite grafts.

A key finding is that scaffold outcomes depend not only on polymer chemistry but also on post-processing, with freeze-drying enhancing porosity and mechanical stability in ways conducive to tissue integration. These insights, obtained in a simplified subcutaneous model, establish a foundation for rational scaffold design prior to vascular implantation studies.

Looking forward, future efforts will focus on fabricating tubular grafts that integrate the strengths of PCL and PLCL, assessing their performance under physiological flow, and scaling coaxial electrospinning processes for translational manufacturing. Together, these steps will advance the development of next-generation AVs shunt grafts with the potential to overcome the limitations of current PTFE conduits.

## Data Availability

All data that support the findings of this study are included within the article (and any supplementary files). Supplementary data 1 available at https://doi.org/10.1088/1748-605X/ae164d/data1.
